# Pathoanatomic Risk Factors for Instability and Adjacent Segment Disease in Lumbar Spine: How to Use Topping Off?

**DOI:** 10.1155/2017/2964529

**Published:** 2017-07-31

**Authors:** J. Bredow, L. Löhrer, J. Oppermann, M. J. Scheyerer, R. Sobottke, P. Eysel, J. Siewe

**Affiliations:** ^1^Center for Spinal Surgery, Schön Klinik Düsseldorf SE & Co. KG, Am Heerdter Krankenhaus 2, 40549 Düsseldorf, Germany; ^2^Department of Orthopedic and Trauma Surgery, University Hospital of Cologne, Cologne, Germany; ^3^Department of Orthopedics, Medical Center City Aachen GmBH, Wuerselen, Germany

## Abstract

**Purpose:**

The goal of this review is to identify criteria indicating implantation of hybrid system into lumbar spine and to evaluate general benefits of use.

**Methods:**

A systematic review of literature was performed using current randomized clinical trials, reviews, and meta-analyses. Data sources included relevant literature of human studies identified through searches of Medline Library until May 2015.

**Results:**

Predisposing factors for Adjacent Segment Disease (ASDi) are discussed in literature: laminar horizontalization, insufficiency of fascia thoracolumbalis, facet tropism, and facet sagittalization. Currently there is no evidence for topping off. There are only 12 studies and these have no consistent statements about use of a hybrid system for avoidance of ASDi.

**Conclusion:**

Hybrid instrumentation of lumbar spine, either with pedicle-based technique or additional spacer, might possibly prevent ASDi from developing in previously damaged segment adjacent to a fusion. Good clinical data proving effectiveness of this new implant technique is as yet unavailable. Thus, currently one must speak of an unevaluated procedure. Various radiological classifications can assist in making a reliable decision as to whether hybrid instrumentation is an appropriate choice of therapy. Pathoanatomical conditions of facet joints and laminae as well as preservation of sagittal balance must also be considered.

## 1. Introduction

Fusion surgery is a standard operative treatment for various pathologies of the lumbar spine, with good clinical results [1]. Overall, however, this procedure is still judged otherwise, even in the literature [2]. Conditions related to the fusion lead to postoperative changes in the biomechanics of the spine. Thus, the initially good clinical results after fusion can be mitigated by degeneration of the adjacent segment [3]. The risk of a clinically relevant adjacent segment disease (ASDi) has been estimated at 0.6–3.9% annually [4, 5]. ASDi are emerging degenerative changes at a spinal level adjacent to a surgically treated level or levels in spine, accompanied by related symptoms like instability, radiculopathy, or myelopathy [6]. Adjacent segment degeneration (ASD) represents radiographic changes without the symptomatology [6].

Risk factors include age at time of surgery (>60 years) and preexisting damage of the facet joints or intervertebral discs in the adjacent segment. Also, multiple segment fusions bordering but not including the L5/S1 segment are more frequently associated with adjacent segment instability. Other operation-specific factors are laminectomy adjacent to a fusion as well as sagittal imbalance [4, 5, 7].

**Figure 1:**
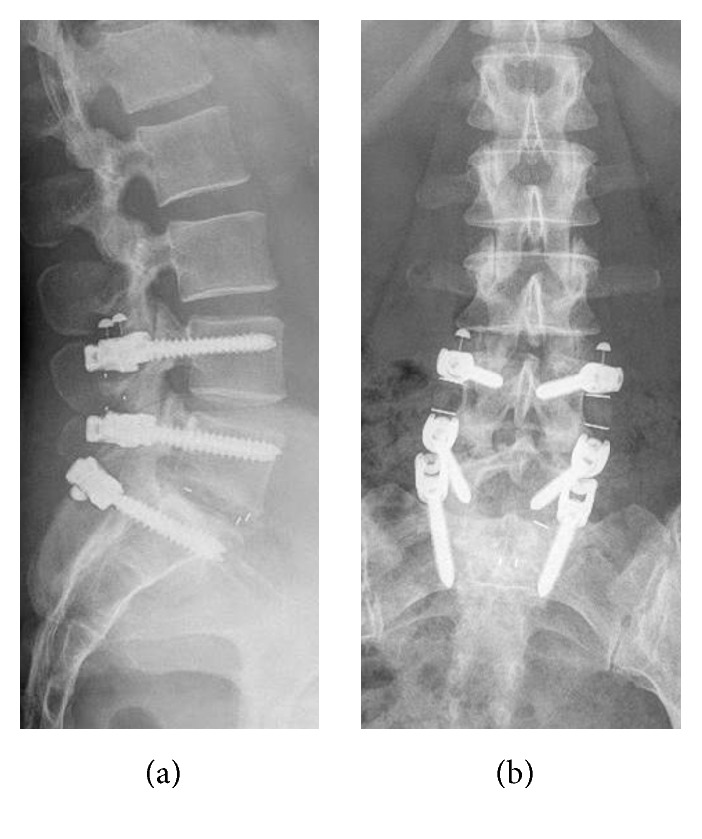
Sagittal (a) and AP (b) radiograph of the lumbar spine in a standing position after hybrid instrumentation with BalanC.

Previous innovations developed to counter the risk of adjacent instability include disc replacement, dynamic stabilization, and percutaneous instrumentation [8]. Posterior dynamic stabilization (PDS) is a rapidly growing field of spine surgery. To simplify the discussion of PDS implants, Khoueir et al. classified these into interspinous spacer, pedicle screw-based, and total facet joint replacement systems [9]. The concept includes conservation and restoration of intervertebral motion in a controlled manner, either through restriction of extreme motion or by limitation of kinetic energy involved in motion. The goal of the implants is to mimic the actions of a healthy spine [9, 10]. The industry has developed various types of pedicle screw rod systems (e.g., Bioflex Spring Rod™, Dynesys®, and IsoBar®). In addition to purely flexible implants, there are various types of flexible pedicle screw rod systems in the form of hybrid systems (“topping off”). This includes the combination of rigid with flexible systems in the region of the segment adjacent to the fusion (e.g., DSS™, DTO™, Dynabolt™, and BalanC™); see Figure 1. Other spine surgeons perform hybrid instrumentation with monosegmental rigid fusion and place an interspinous spacer in the adjacent segment [11]. To date, there is no evidence that these systems provide clinical benefits to patients, and long-term results are needed to support their use [8].

The goal of this review is to identify criteria that indicate implantation of a hybrid system into the lumbar spine and to evaluate general benefits of use at the current time. To accomplish this, the available studies investigating pedicle-based hybrid systems will be systematically evaluated. In addition, pathoanatomic factors indicating use of such systems or influencing the development of ASD should be identified. Finally, based on these results, potential criteria indicating the use of hybrid instrumentation will be formulated.

## 2. Materials and Methods

We conducted a systematic Medline database search for literature regarding hybrid instrumentation (e.g., Figure 1) up to and including August 2015. The search was limited to human studies in the English language. The following search terms were used:

- Topping off + lumbar spine
- Hybrid instrumentation + lumbar spine
- Dynamic stabilization + lumbar spine
- Adjacent segment disease + lumbar spine
- Adjacent segment degeneration + lumbar spine.

Exclusion criteria were tumors, infections, and treatment for trauma or congenital deformities. Similarly, biomechanical studies as well as nonhuman in vivo or in vitro studies were excluded. The bibliographies of the searched articles were also systematically evaluated to identify further articles.

Thus, all studies discussing the general use of these systems were collected. In addition, all articles were further evaluated as to whether concrete indication criteria could be identified.

A second search identified articles published up to August 2014 in the Medline database dealing with the development of segment degeneration based on pathoanatomic conditions (facet sagittalization, facet tropism, and laminar horizontalization). The search was again limited to human studies in the English language. Here, too, bibliographies of the articles were systematically examined. The goal was the identification of pathoanatomic situations that might favor the development of segment degeneration. Investigations with fewer than ten subjects *n* < 10 as well as animal, cadaver, and biomechanical studies were excluded.

**Figure 2:**
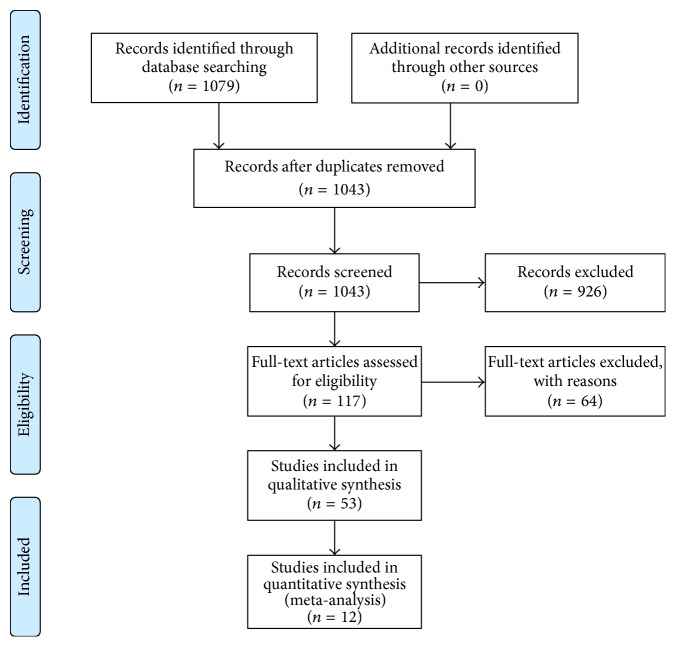
Flow chart systematic literature search hybrid instrumentation.

We worked according to the PRISMA criteria and carried out the literary research with 2 persons. The search was performed exclusively in the Medline database. The evidence level was determined by the two reviewers according to the Agency for Healthcare Research and Quality. The search algorithm is presented according to the PRISMA criteria in Figure 2.

### 2.1. Data Extraction

The following data were collected from the included articles: study design, intervention, subject number, follow-up interval, age, specific inclusion/exclusion criteria for supplemental dynamic hybrid instrumentation, outcome parameters, complications, occurrence of ASD or ASDi, and summary.

## 3. Results

### 3.1. Hybrid Instrumentation

#### 3.1.1. Study Selection

The Medline database search yielded 1097 articles.

The initial evaluation of titles and/or abstracts left 117 articles. After reading the entire texts, 12 studies were included in the study (Table 1).

All studies (Figure 2) were published after the year 2007. Various pedicle-based hybrid implants (Dynesys, Agile Topping off, IsoBar, NFLex, Flex Plus, Bioflex, and Dynesys to Optima (DTO)) were tested.

The number of test subjects was between 15 and 46, with follow-ups ranging from 9 to 76 months.

The nonrandomized work of Zagra et al. compared dynamic versus hybrid implants. There was no standard control group in this study. 32 patients were evaluated over a period of 12 months. There were significant reductions in the Oswestry Disability Index (ODI) as well as postoperative complaints using a visual analog scale (VAS); however, 12.5% of subjects showed no clinical improvement. In fact, over the course of the study, 6.3% of the subjects required implant removal due to substantial clinical deterioration [21].

Similarly, in 2008, Kumar et al. [19] showed a significant reduction of the clinical Woodend scores. However, it was noted that the degenerative disc disease progressed regardless of the dynamic implant.

The study from Ogawa et al. in 2009 [13] found no difference in clinical outcomes between those using a sublaminar wiring stabilization system and the control group. They did, however, notice an improvement in lordosis of the hybrid group. It is assumed that the sublaminar wiring stabilization system significantly reduced the range of motion of the adjacent segment, thus maintaining the sagittal profile and resulting in prevention of ASD.

In our view, the most important investigations were carried out by Fu, Maserati, and Putzier [15, 17, 23].

In 2010, Maserati et al. [17] examined 24 patients who were treated with the DTO system from Zimmer. In this group, the VAS scores were reduced. The observed complications were all independent of the implanted instrumentation. Thus, for the first time there was an overall positive result for the technique, although the authors considered further studies with longer follow-up necessary for confirmation.

Similarly in 2010, Putzier et al. [15] published a prospective, randomized investigation in which the Allospine Dynesys System (Zimmer) was compared to standard PLIF. For the first time, the authors based the inclusion criteria on the extent of degeneration (the Modic signs were determined in the fusion segment). This study design, which we consider the best to date, observed 60 patients over 72 months. The VAS and ODI were significantly reduced in both groups. Reduced ASD progression was also observed. Finally, the authors offered no recommendations regarding the use of the implant, because less progression of ASD was matched by a higher rate of implant failure.

In 2014, Fu et al. [23] published data using the IsoBar system. In this prospective study of 36 subjects observed over 2 years, there were no complications. In addition, again significant reductions in ODI and VAS were documented. However, degenerative disc disease progressed further regardless of the dynamic system.

### 3.2. Pathoanatomic Risk Factors for the Development of Segment Degeneration

#### 3.2.1. Study Selection

**Figure 3:**
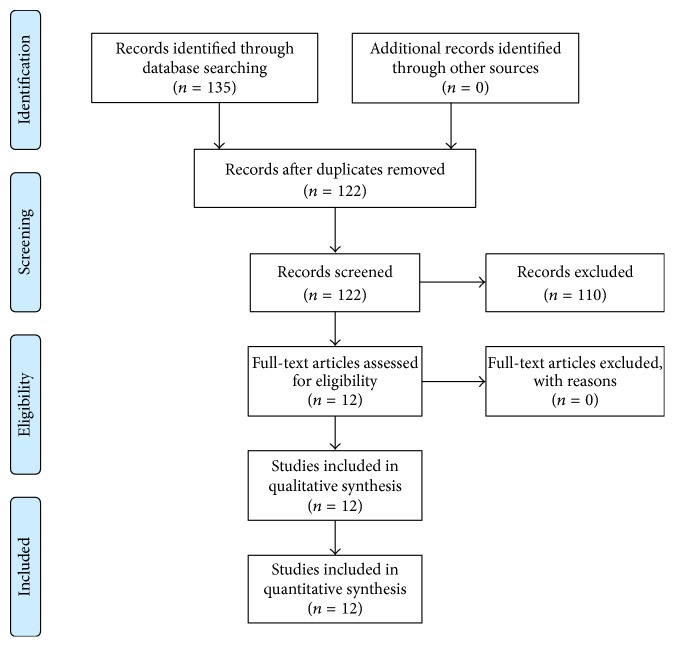
Flow chart systematic literature search for pathoanatomic risk factors.

The database search yielded a total of 135 studies, from which exclusion of duplicate citations yielded 122 articles. 110 of these articles were excluded because they did not offer information relevant to our questions. Twelve articles remained for full-text checking regarding suitability. From these, none were excluded and, thus, twelve items were included in the review (Figure 3).

#### 3.2.2. Study Characteristics

The twelve studies identified during the literature search were published more recently than 2008 with one exception (Okuda et al., 2004, [24]). These were mostly retrospective studies, some of which with case-control design. The studies focused on patient collectives between 20 and 109 patients. The average was 62 subjects. Follow-up ranged from 39.3 to 134 months. Average patient age was between 48.5 and 65.4 years.

The studies each identified various factors as the cause for ASD (see Table 2).

In 2008, in a retrospective case-control study of 20 patients with 42-month follow-up comparing the original posterior lumbar intervertebral fusion (PLIF) of L4/5 and reoperation with PLIF L3/4 or decompression, Okuda et al. concluded that laminar horizontalization of 130° accelerates ASD [25].

Analyzing the data of 45 patients over 71 months in a retrospective case-control study, Rothenfluh et al. identified pelvic incidence greater than 10° as an ASD accelerating factor. However, the intervention was inconsistent, with PLIF in one to three segments between L2 and S1 [26].

Jeong et al. found that insufficiency of the tight thoracolumbar fascia is associated with the development of ASD. This study was a retrospective evaluation of 68 patients and used morphologic changes of the TLF in postoperative magnetic resonance imaging to investigate [27].

In the prospective study of 68 patients by Anandjiwala et al., preoperative disc degeneration of the adjacent segment was discussed as a preexisting factor influencing disease development. The postoperative observation period after decompression and lumbar or lumbosacral fusion averaged 67.4 months [28].

Hikata et al. performed a retrospective study investigating the postoperative course of 54 patients after PLIF L4/5 over 55 months, some of whom underwent decompression of L3/4. They concluded that facet joint sagittalization (decreasing convergence of the facet joints in the axial plane) from 52.7 to 67.1° can lead to subsequent development of anterolisthesis with concomitant adjacent instability [29].

Bae et al. postulated that a loss of lumbar segmental lordosis promotes ASD. They found this after retrospective analysis of data from 103 patients collected after fusion surgery over an average of 59.2 months [30].

Chen et al. were unable to determine any pathoanatomic factors. However, in a retrospective study of 109 patients over 39.3 months, age of the patient at the time of PLIF L4/5 surgery was implicated for the development of ASD [31].

**Figure 4:**
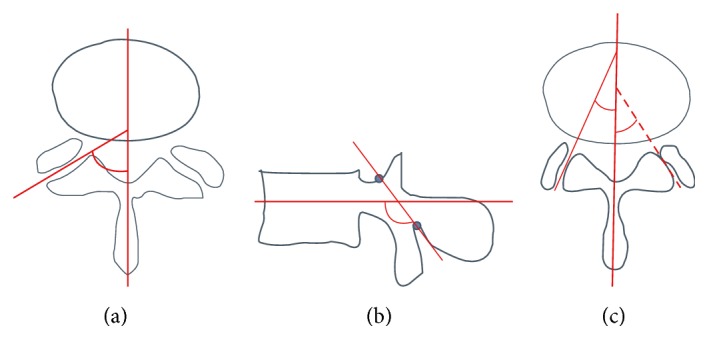
Pathoanatomic risk factors for ASD in lumbar spine are (a) facet sagittalization < 52.7°, (b) laminar horizontalization > 130°, and (c) facet tropism (more than 10° divergence of both facet joints).

In addition to laminar horizontalization of >130°, Okuda et al. found the coexistence of facet tropism (Figure 4) to be a risk factor for ASD [25]. They carried out a retrospective study [24] of 87 patients in 2004, in which data over a postoperative course of 43 months after PLIF L4/5 was analyzed.

Three studies identified preoperative damage to the facet joint as a cause or risk factor for ASD [32–34].

According to Yu et al., spondylolytic spondylolisthesis of fused segment is not a risk factor for ASD development. They performed a retrospective analysis of data from 63 patients followed up for an average of 43.1 months after PLIF with posterior decompression [35].

## 4. Discussion

### 4.1. Hybrid Instrumentation

In summary, there is currently no conclusive evidence in the literature regarding the use of hybrid instrumentation. There are only twelve existing studies that investigate the use of “topping off,” and these make no uniform statements regarding the use of diverse systems. Although almost all of the studies have shown significant reductions in pain or established spine scores [12–17, 19–23], comparable observations could also be raised in the context of standard procedures.

The analyzed studies are thus in agreement that the reduced risk of degeneration of the disc or the adjacent segment through use of dynamic topping off implants is accompanied by increased risks of implant failure.

It is crucial, however, to challenge the study inclusion criteria constructed by us for this review study. Thus, the inclusion criteria of study subjects are nonuniform and mostly widely disbursed.

Various prognostic factors were also not considered. For example, the segment superior to the planned fusion should be intact to avoid protracted degeneration. Here, most studies are lacking a standardized collection of independent criteria, for example, the Modic changes as described in the studies by Putzier et al. (in the fusion segment) [15] and Siewe (in the fusion segment plus the adjacent segment) [36, 37].

In summary, it seems clear that as yet there is insufficient evidence validating the use of dynamic implants and that, thus, the procedure should be performed within the context of clinical trials.

### 4.2. Pathoanatomic Risk Factors for the Development of Segment Degeneration

In the reviewed works, pathoanatomic conditions increasing the risk of segment degeneration, particularly after fusion, were identified. Despite the indications for predisposing factors, the evidence in this regard is weak. Thus, laminar horizontalization over 130°, facet sagittalization between 52.7 and 67.1°, and facet tropism of over 10° are considered highly predisposing for ASD (Table 2). The described pelvic incidence of more than 10° as an accelerating factor for ASD [26] does not seem coherent. Given the fact that most of patients have a pelvic incidence of more than 10°, this possibility is excluded as a predicting factor. Similarly, the operation-related insufficiency of the thoracolumbar fascia is a focus of discussion. This has so far not been sufficiently quantified [24, 38–50]. Just as well the loss of lumbar segmental lordosis can not be quantified so far.

As already mentioned, laminar horizontalization is a possible risk factor for degenerative spondylolisthesis. This correlation of horizontalization of the lamina as well as the facet joints was observed in cases developing degenerative spondylolisthesis by Nagaosa et al. [24, 48].

Sagittalization of the facet joints in segment L4-5 was also more frequently observed in patients with degenerative spondylolisthesis compared to normal patients and patients with isolated spinal stenosis [47]. In addition to this highly significant observation in segment L4/5, patients with degenerative spondylolisthesis showed similarly significant sagittalization of the facet joints in all other segments. In a study from Boden et al., the average angle of the facet joints in asymptomatic volunteers was 41° (range 37.6°–44.6°). In patients with degenerative spondylolisthesis, the average of 60° (range 52.7°–67.1°) was markedly higher. In this study, individuals in whom the facet joint angles of segment L4/L5 were greater than 45° bilaterally were 25 times more likely to suffer a degenerative spondylolisthesis than the control group [38].

In addition to the sagittalization, asymmetry of the facet joints (facet tropism) has been discussed as leading to abnormal rotation of the segment and increased torsion on the disc, leading to subsequent progressive degeneration. However, study positions on this are very heterogeneous [24, 38–47]. Also, the influence of facet tropism on the occurrence of disc herniation as well as herniation in childhood and adulthood is also under discussion [43, 49]. Symptomatic patients with lumbar disc herniation show a median facet tropism of 10.3° in segment L4-L5 and a control group a deviation of only 5.4° (*p* = 0.05) [38].

Particularly, the coexistence of a laminar horizontalization of L3 and a facet tropism of L3-L4 is considered a risk factor for degeneration of L3-L4 after PLIF of L4-L5 [24]. Considering this, these conditions would particularly suggest the use of hybrid instrumentation of L3-L4 for a planned L4-L5 fusion.

#### 4.2.1. Possibilities for the Use of Topping Off

**Figure 5:**
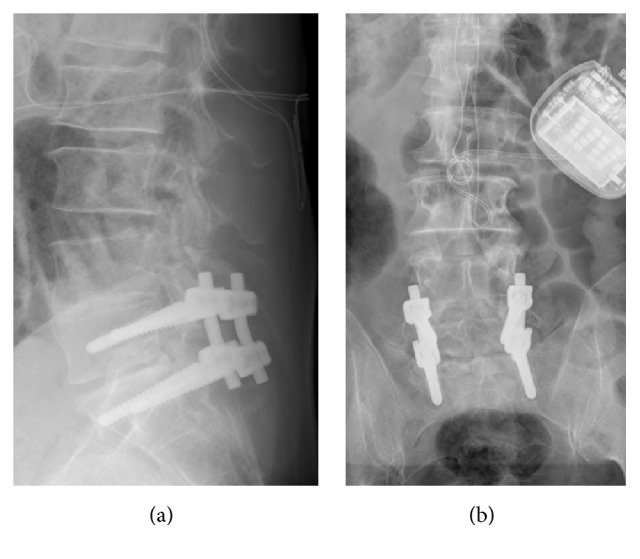
Sagittal (a) and AP (b) radiograph of the lumbar spine in a standing position after posterior lumbar interbody fusion in L5 and S1 with ASD in L4/5.

**Figure 6:**
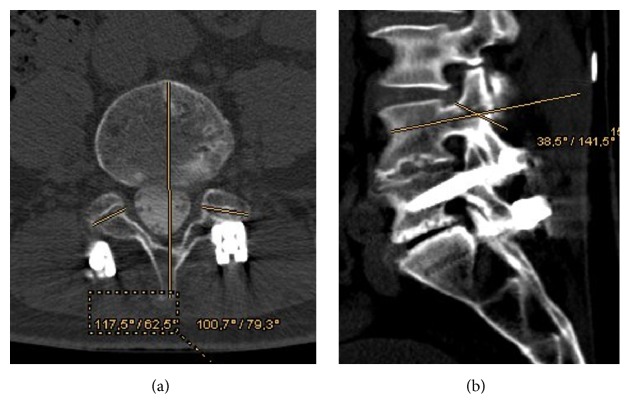
CT-scan of patient in Figure 5 with posterior lumbar interbody fusion in L5/S1 and ASD in L4/5. (a) Axial slice with facet tropism (17°) and (b) sagittal slice with laminar horizontalization of 141,5°.

Basically, at the present time there is no clear evidence indicating routine benefits of hybrid instrumentation. In general, from the authors' view the following indication possibilities are yielded from clinical experience and must be evaluated in future clinical studies:

1. Primary treatment: in the case of spondylodesis, primary implantation of pedicle-based hybrid implants is performed to reduce the risk of ASD in cases of preexisting degeneration of the adjacent segment.
2. When considering use of an additional interspinous spacer, the anatomic structures of the facet joint should be taken into account. Interspinous spacers can be applied in cases of convergent facet joints. In cases of increasingly sagittally inclined facet joints or laminae, pedicle-based hybridization instrumentation is preferable.
3. If ASDi has already occurred during the postspondylodesis follow-up (Figure 5), there is the possibility to extend the fusion with additional hybrid instrumentation superiorly to prevent renewed adjacent segment degeneration. Nevertheless sagittal imbalance must be addressed.
4. Postfusion, complete laminectomy, and the associated resection of the longitudinal ligament for reconstruction of the tension band or after the undercut decompression superiorly with compromise of the upper facet joints: laminectomy adjacent to a fusion is a risk factor for ASDi [4].
5. The pathoanatomic parameters listed above (Table 2) favoring ASD or ASDi should be taken into consideration. In cases where there is thoracolumbar fascia insufficiency, facet sagittalization of 52.7 to 67.1°, and particularly facet tropism of over 10° or laminar horizontalization of over 130°, according to current perspectives, topping off could be indicated (Figure 6).

*The Pfirrmann Classification on MRI [50]*. Disc degeneration should be present, which does not exceed a certain grade, since otherwise this would also approach an indication for fusion (e.g., Pfirrmann grades 2–4).

*Fujiwara Classification on MRI [51]*. Evaluation of facet joint arthritis on MRI. The facet joint arthritis should not be maximally pronounced in the segment to be flexibly instrumented, since again this suggests an indication for fusion (e.g., Fujiwara type II).

### 4.3. Contraindications

In general, the preexistence of instability is a contraindication for flexible instrumentation of a segment. Several criteria must be used for the diagnosis of segment instability, which have been variably defined in the literature.

Possible definition criteria could be spondylolisthesis > 4 mm, segmental kyphosis > 10°, rotational hypermobility > 15° (functional projection), lateral translation > 3 mm (AP projection), and disc wedging > 5 (A.P. projection) [36, 37].

## 5. Conclusion

Hybrid instrumentation of the lumbar spine, either with pedicle-based technique or with additional spacer, might possibly prevent ASD from developing in a previously damaged segment adjacent to a fusion. Good clinical data proving the effectiveness of this new implant technique is as yet unavailable. Thus, currently one must speak of an experimental procedure. Various radiological classifications can assist in making a reliable decision as to whether hybrid instrumentation is an appropriate choice of therapy. The pathoanatomical conditions of the facet joints and the laminae as well as the preservation of sagittal balance must also be considered. This manuscript should help to define indications for hybrid instrumentation in lumbar spine surgery.

## Figures and Tables

**Table 1:** Overview of pedicle-based study methods.

Author	Implant	Subject number	Follow-up (months)	Publication year	Level of evidence	Study design
Kim et al. [12]	Bioflex	46	9	2007	2b	Retrospective
Ogawa et al. [13]	Sublaminar wiring	54	40	2009	1b	Prospective
Kaner et al. [14]	Agile topping off	15	19	2009	1b	Prospective
Putzier et al. [15]	Dynesys transition	22	76	2010	1b	Randomized
Schwarzenbach et al. [16]	Dynesys	31	39	2010	3b	Retrospective
Maserati et al. [17]	Dynesys to Optima	22	(1–22)	2010	2b	Retrospective
Hudson et al. [18]	IsoBar	22	24	2011	1b	Prospective
Kumar et al. [19]	Dynesys	32	24	2012	1b	Prospective
Coe et al. [20]	NFlex	40	24	2012	2b	Retrospective
Zagra et al. [21]	FlexPlus	32	12	2012	1b	Prospective
Li et al. [22]	IsoBar	36	24	2013	2b	Retrospective
Fu et al. [23]	IsoBar	36	24	2014	1b	Retrospective

**Table 2:** Predisposing pathoanatomic factors for ASD.

(i) Laminar horizontalization > 130°
(ii) Thoracolumbar fascia insufficiency
(iii) Facet sagittalization 52.7 to 67.1°
(iv) Loss of segmental lordosis
